# Hemagglutinin Quantitative ELISA-based Potency Assay for Trivalent Seasonal Influenza Vaccine Using Group-Specific Universal Monoclonal Antibodies

**DOI:** 10.1038/s41598-019-56169-5

**Published:** 2019-12-23

**Authors:** Wonil Chae, Paul Kim, Hanna Kim, Yu Cheol Cheong, Young-Seok Kim, Sang Moo Kang, Baik L. Seong

**Affiliations:** 1 Department of Biotechnology, College of Life Science and Biotechnology, Yonsei University, Seoul, Republic of Korea; 2 Vaccine Translational Research Center, Yonsei University, Seoul, Republic of Korea; 3 Department of Integrated OMICS for Biomedical Science, College of World Class University, Yonsei University, Seoul, Republic of Korea; 4 Center for Inflammation, Immunity & Infection, Institute for Biomedical Sciences, Georgia State University, Atlanta, GA USA

**Keywords:** Assay systems, ELISA, Vaccines

## Abstract

The assurance of vaccine potency is important for the timely release and distribution of influenza vaccines. As an alternative to Single Radial Immunodiffusion (SRID), we report a new quantitative enzyme-linked immunosorbent assay (ELISA) for seasonal trivalent influenza vaccine (TIV). The consensus hemagglutinin (cHA) stalks for group 1 influenza A virus (IAV), group 2 IAV, and influenza B virus (IBV) were designed and produced in bacterial recombinant host in a soluble form, and monoclonal antibodies (mAbs) were generated. The group-specific ‘universal’ mAbs (uAbs) bound to various subtypes of HAs in the same group from recombinant hosts, embryonated eggs, and commercial vaccine lots. The calibration curves were generated to assess the sensitivity, specificity, accuracy, and linear dynamic range. The quantitative ELISA was validated for the potency assay of individual components of TIV- H1, H3, and IBV- with good correlation with the SRID method. This new assay could be extended to pandemic or pre-pandemic mock-up vaccines of H5 of group 1 and H7 virus of group 2, and novel HA stalk-based universal vaccines.

## Introduction

Since the first influenza vaccine was introduced in 1942^1^, various types of vaccine formulations have been developed. The trivalent influenza vaccine containing two strains of influenza A virus (IAV) and one strain of influenza B virus (IBV) has been distributed since 1978^1^. Recently, a quadrivalent vaccine has been recommended to provide protection against two co-circulating lineages of IBV^2^. In addition, the recombinant protein-based quadrivalent vaccine has also been licensed and distributed^3^. Furthermore, ‘universal’ vaccines that would provide protection against various drift or potential pandemic strains of viruses are being actively researched^4–6^.

The WHO guidelines specify that the manufacturers determine the potency of the vaccines at the time of release^7^. The single radial immunodiffusion assay (SRID), based on the immunological reaction between antisera and test hemagglutinin (HA) antigen, has been used as a golden standard potency assay for seasonal vaccines since the 1970s^8^. As the only internationally accepted assay for potency and stability, SRID is labor-intensive, relatively insensitive, not amenable to automation, and therefore, time-consuming. Requirement of seasonal reference reagents further necessitates complex interactions among vaccine producers, surveillance laboratories, and regulatory agencies. Importantly, the requirement of strain-specific reference antigen and anti-serum is a major limitation that might be a hindrance to a timed supply of the vaccine, as exemplified by the 2009 swine flu (H1N1) pandemic^9^. Therefore, there is a need for developing and testing alternative potency assays.

In this study, we developed a new quantitative Enzyme-Linked Immunosorbent Assay (ELISA) for testing the trivalent seasonal influenza vaccine. The consensus HA (cHA) stalk for group 1 influenza A virus (IAV), group 2 IAV, and influenza B virus (IBV) were designed and successfully produced in a bacterial recombinant host as soluble form. Monoclonal antibodies (mAbs) that bind to HAs of various subtypes and drift strains within the same group were generated. The group-specific ‘universal’ mAb (uAb)s bind to various subtypes of HAs including recombinant HA, egg-derived HA, and commercial vaccine antigens in the same group. The HA quantitative ELISA for trivalent influenza vaccine using uAbs was validated for the potency assay of the trivalent vaccine, comprised of H1N1 (group 1 IAV), H3N2 (group 2 IAV), and IBV, with good correlation with the SRID-based method. The impact of the present assay could be far-reaching; in addition to seasonal vaccines, the same assay platform could be further extended to potential pandemic vaccines of H5N1 virus of group 1 and H7N9 virus of group 2^10,11^, and for HA stalk-based universal influenza vaccines^12–14^.

## Results

### Development of consensus hemagglutinin stalk

**Figure 1:**
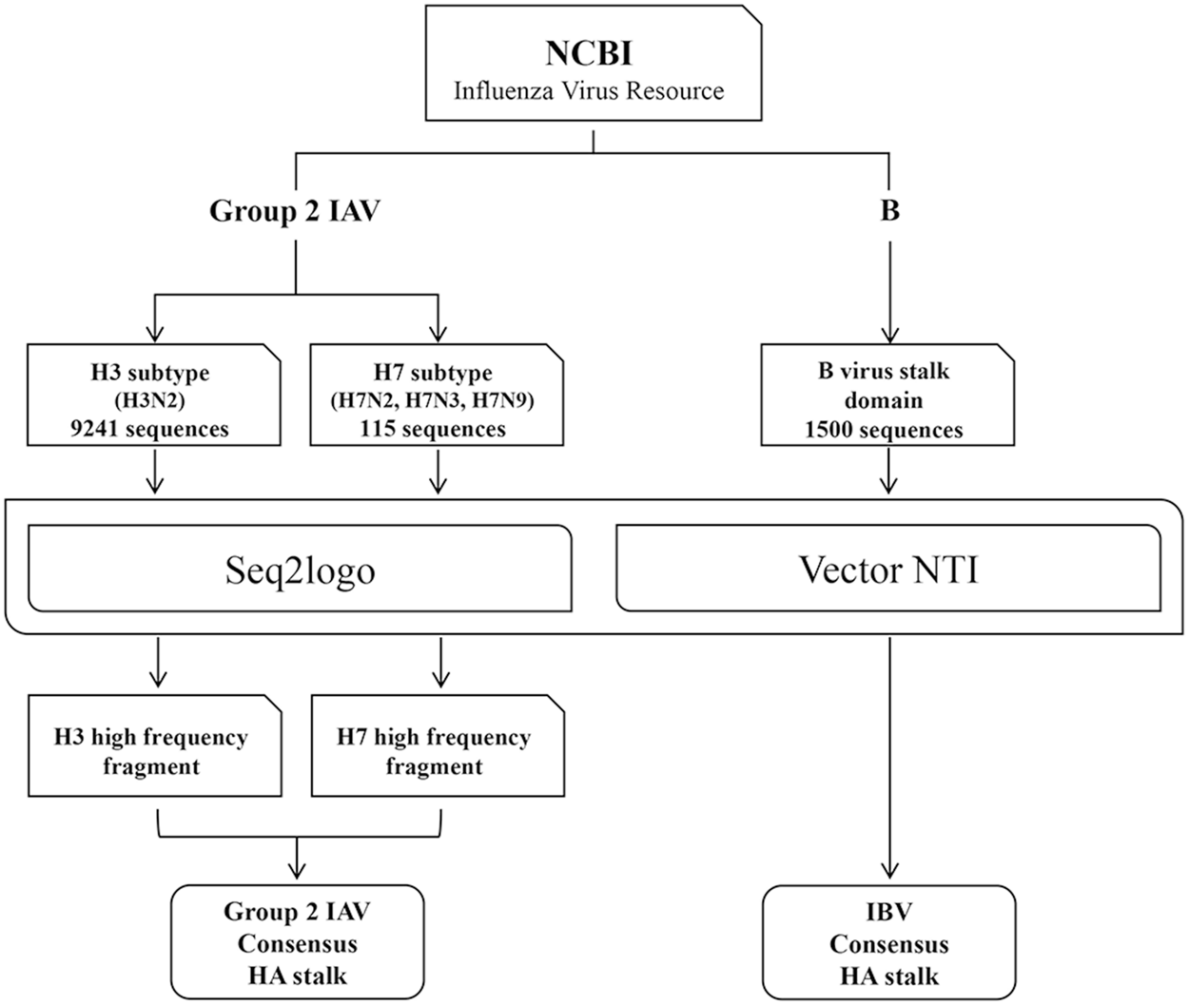
Generation of consensus hemagglutinin(cHA) stalk antigen. Schematic diagram of methods used for deducing cHA stalk sequence from the influenza HA sequence library.

The consensus sequences of hemagglutinin (HA) stalk was deduced from the HA sequence library^15^. The number of reference HA sequences and scheme for cHA sequence design are described in Fig. 1. The cHA stalk for group 1 IAVs was designed and validated previously^15^. The cHA stalk for group 2 IAVs was generated based on H3 and H7 high frequency fragments consisting of the most conserved amino acid at each residue (Supplementary Fig. 1a). In case of the cHA stalks for IBV, the sequence was deduced directly without recourse to high frequency fragment, especially because IBVs are classified into only two lineages, Yamagata-like and Victoria-like, in clear contrast to IAVs which are classified into various (total 17) subtypes. Furthermore, referenced HA stalk sequences of IBVs showed extremely high (about 98%) similarity in the stalk regions (data not shown). The computationally designed cHA stalk sequences for group 2 IAVs and IBV are shown in Supplementary Fig. 1b.

The secondary structure prediction^16,17^ confirmed that the cHA stalk of group 2 IAVs and IBV adopt structural patterns similar to that of HAs of natural isolates (Supplementary Fig. 2), suggesting that the designed cHA stalks are suitable for anti-stalk universal antibody generation. The cHA stalks were genetically fused with the N-terminal RNA interaction domain of lysyl tRNA synthetase of murine origin (mRID)^15,18^. Consistent with and further extending the chaperone (RNA as chaperone) function^18^, the mRID-cHA stalks were successfully expressed in *E. coli* as soluble form (Supplementary Fig. 3a,d) and purified by one-step Ni+ affinity chromatography (Supplementary Fig. 3c,e).

### Validation of universal antibodies

The uAbs for group 2 IAV and IBV cHA stalk were produced by hybridoma technology^19^. Positive clones were screened by ELISA using mRID-cHA stalk as the coating antigen. ‘4F11’ and ‘10F8’ clones were identified as uAbs for group 2 IAVs and IBVs, respectively. The uAbs were tested by indirect ELISA with various HAs to validate universal binding to group-specific HA antigens. Moreover, statistical analysis was conducted based on the ELISA results to assess statistical indicators in terms of linearity, sensitivity, and repeatability, to validate their potential of the reagents as references for HA quantification.

The uAbs were designed to target HA stalk domain which is immunologically subdominant and structurally shielded by the HA globular domain (or HA1 subunit)^20^. Thus, the HAs were pretreated with pH 4.5 NaOAc buffer containing 200 mM DTT^15^ to enhance binding of the antibody by induction of pH dependent conformational changes^21^ and disruption of disulfide bonds^22^.

**Figure 2:**
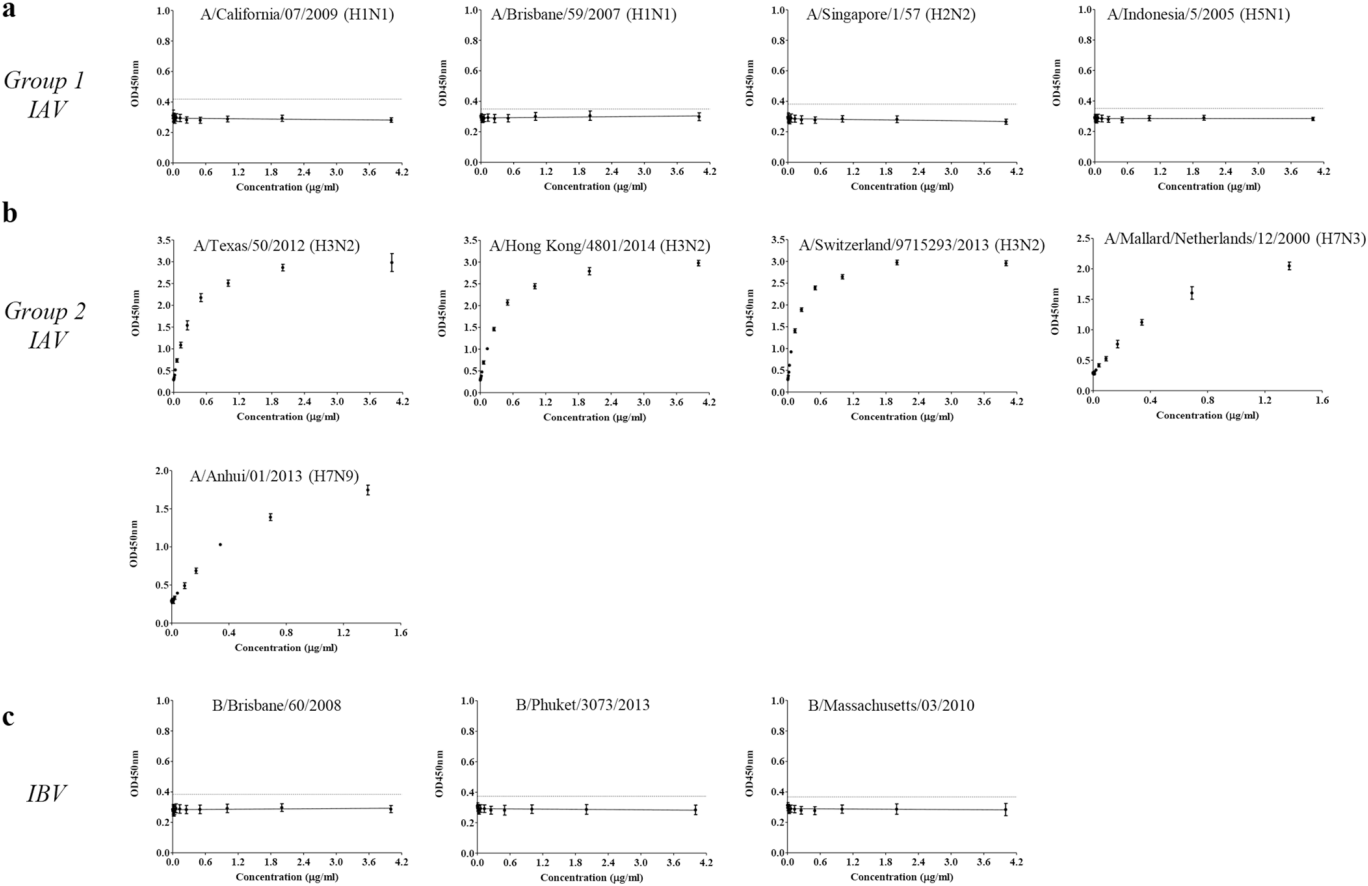
Evaluation of group 2 IAV universal antibody 4F11 with egg derived HAs. Group-specific universality of 4F11 was validated by ELISA with egg derived HAs. Error bars indicate standard deviation across 5 replicates. Dotted lines indicate limit of detection (LOD = Mean_(PBS)_ + 3 SD_(PBS)_). (**a**) ELISA with HAs from group 1 IAVs. (**b**) ELISA with HAs from group 2 IAVs. (**c**) ELISA with HAs from IBV.

First, the uAbs were tested with standard HAs from NIBSC, which are egg-derived reference reagents for SRID. The 4F11 bound to HAs of various subtypes belonging to group 2 IAVs (three different strains of H3N2 and two different H7 subtypes, H7N3 and H7N9). However, it did not bind with HAs of group 1 IAV (H1N1, H2N2, H5N1) or with IBVs of Yamagata-like and Victoria-like lineages, confirming the group 2 specificity (Fig. 2). The ELISA response to the HAs from group 2 IAVs was highly correlated with the HA concentrations (average Coefficient of determination, R^2^ = 0.997 ± 0.002). Also, the sensitivity of 4F11 to the various HAs was significantly high (average Limit of Detection, LOD ≤0.017 μg/ml). In addition, the ELISA results showed high repeatability (average % Constant of Variation, CV = 5.074 ± 0.578). Detailed results are described in Table 1. The results confirmed 4F11 as the uAb for the specific detection of HAs of group 2 IAVs, including H3N2 component in the seasonal influenza vaccine.

**Table 1:** Validation of linearity, sensitivity, and reproducibility of the ELISA with egg derived HAs.

Egg-derived HA (NIBSC)			uAb (group 2 IAV)				uAb (IBV)			
			4F11				10F8			
Type	HA subtype	Strain	Response	Linearity (R^2^)	Sensitivity (LOD (µg/ml))	Reproducibility (%CV)	Response	Linearity (R^2^)	Sensitivity (LOD (µg/ml))	Reproducibility (%CV)
Group 1 IAV	H1	A/California/7/09 (NYMC-X181)	—				—			
		A/Brisbane/59/2007 (IVR-148)	—				—			
	H2	A/Singapore/1/57	—				—			
	H5	A/Anhui/1/05 IBCDC-RG-6	—				—			
Group 2 IAV	H3	A/Hong Kong/4801/2014 (NYMC X-263B)	+	0.995	0.011	4.913	—			
		A/Switzerland/9715293/2013 (NIB88)	+	0.996	0.004	4.191	—			
		A/Texas/50/2012 (NYMC X-223A)	+	0.996	0.010	5.800	—			
	H7	A/mallard/Netherlands/12/2000	+	0.999	0.011	4.856	N/A			
		A/Anhui/1/2013	+	0.999	0.017	5.610	N/A			
IBV	Yamagata	B/Phuket/3073/2013	-				+	1.000	0.004	3.799
		B/Massachusetts/02/2012	—				+	0.993	0.006	6.250
	Victoria	B/Brisbane/60/2008 (NYMC BX-35)	—				+	0.980	0.000*	5.287
		B/Maryland/15/2016	N/A				+	0.994	0.002	6.098

*0.000 means that the value is lower than 0.001.

Response: ‘+’ (positive); ‘−’ (negative).

N/A: Not available.

Calibration curves determined by four-parameter linear regression. R^2^ (Coefficient of determination), LOD (Limit of detection), % CV (% Constant of variation).

**Figure 3:**
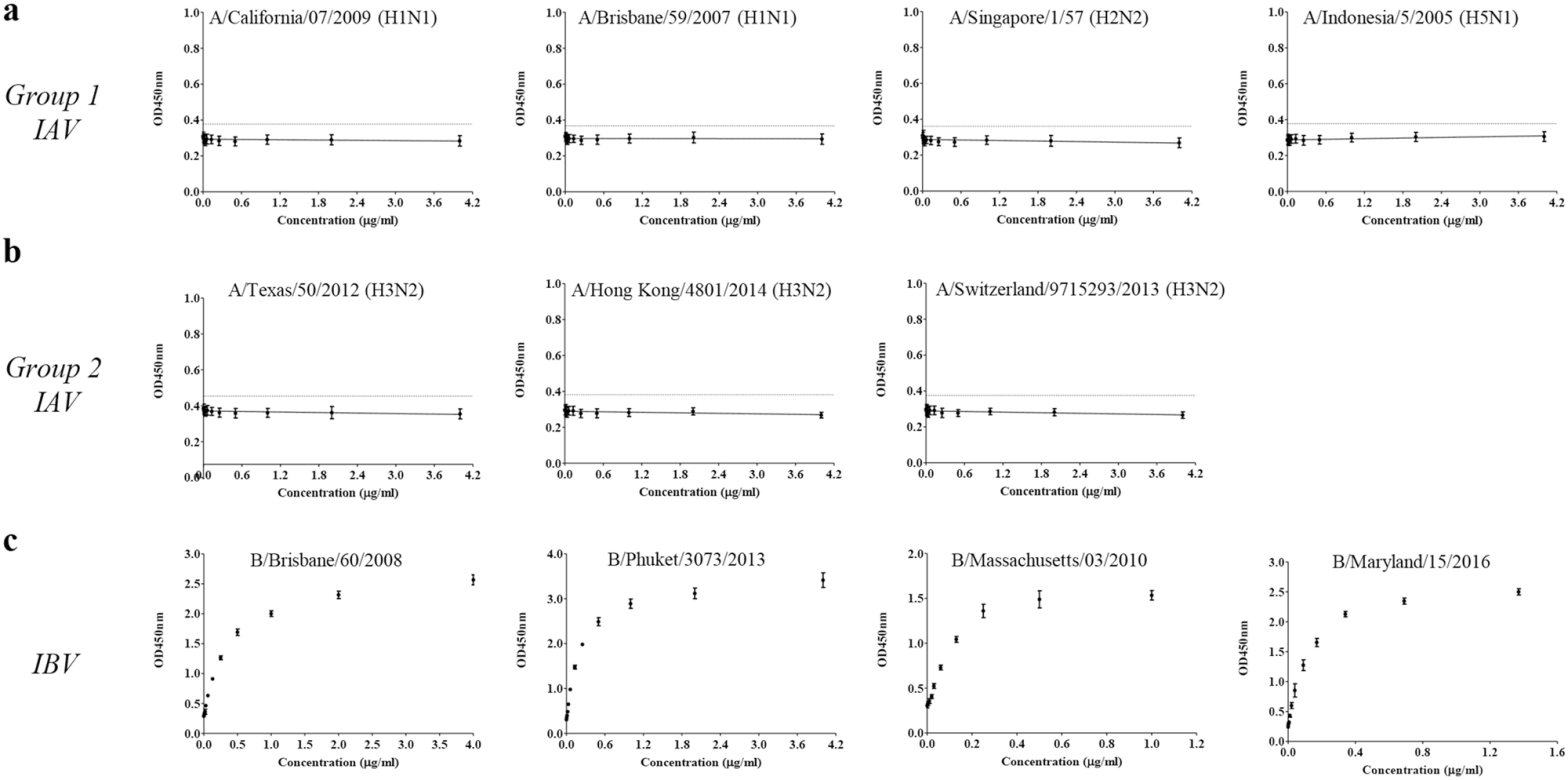
Evaluation of IBV universal antibody 10F8 with egg derived HAs. Group-specific universality of 10F8 was validated by ELISA with egg derived HAs. Error bars indicate standard deviation across 5 replicates. Dotted lines indicate limit of detection (LOD = Mean_(PBS)_ + 3 SD_(PBS)_). (**a**) ELISA with HAs from group 1 IAV. (**b**) ELISA with HAs from group 2 IAV. (**c**) ELISA with HAs from IBV.

Likewise, the 10F8 clone, universally bound to the HAs of IBV of both Yamagata (B/Phuket/3073/2013 and B/Massachusetts/03/2010) and Victoria lineages (B/Brisbane/60/2018 and B/Maryland/15/2016) but failed to bind to HAs of IAVs (Fig. 3). There was strong positive correlation between the ELISA response and the concentrations of HAs (average R^2^ = 0.992 ± 0.007). Also, the 10F8 showed high sensitivity (LOD ≤0.006 μg/ml) and the ELISA results showed high repeatability (average % CV = 5.359 ± 0.972) as described in Table 1. The results confirm that 10F8 is the uAb for the specific detection of HAs of IBVs, as a component of trivalent seasonal influenza vaccine.

Furthermore, the uAbs were tested with mammalian-derived recombinant HA (rHA) (Sino Biological, Beijing, China) to validate if uAbs can be used for the quantitation of HAs produced from recombinant hosts. The 4F11 showed universal binding to the HAs from group 2 IAVs but failed to bind HAs from group 1 IAVs or IBVs (Supplementary Fig. 4). The results confirm the specificity of 4F11, further extending the results obtained with egg-derived HAs (Fig. 2). The ELISA responses were in good correlation with the rHA concentrations (average R^2^ = 0.998 ± 0.002). Also, 4F11 exhibits high sensitivity to various rHAs (LOD ≤ 0.016 μg/ml) and the ELISA results were highly reliable (average % CV = 3.962 ± 0.748). Detailed results are described in Table 2. The 10F8 successfully bound to the rHAs of both B/Yamagata/16/88 and B/Massachusetts/03/2013, produced from insect cell recombinant hosts (Supplementary Fig. 5). The results showed that 10F8 could universally bind to the HAs of IBVs irrespective of its lineage without cross reactivity to those from IAVs. The ELISA responses were highly correlated with the HA concentrations (R^2^ for B/Yamagata/16/88 = 0.991, R^2^ for B/Massachusetts/03/2012 = 0.993). Also, the 10F8 showed high sensitivity (LOD ≤ 0.141 μg/ml) and the ELISA results were high reliable (average % CV = 4.972 ± 0.141). Detailed results are described in Table 2.

**Table 2:** Validation of linearity, sensitivity, and reproducibility of the ELISA with mammalian derived HAs.

Mammalian derived HA			uAb (group 2 IAV)				uAb (IBV)			
			4F11				10F8			
Type	HA subtype	Strain	Response	Linearity (R^2^)	Sensitivity (LOD (µg/ml))	Reproducibility (%CV)	Response	Linearity (R^2^)	Sensitivity (LOD (µg/ml))	Reproducibility (%CV)
Group 1 IAV	H1	A/California/07/2009	—				—			
	H2	A/Canada/720/2005	—				—			
	H5	A/Indonesia/5/2005	—				—			
Group 2 IAV	H3	A/Texas/50/2012	+	0.997	0.011	2.924	—			
		A/Brisbane/10/2007	+	1.000	0.008	4.307	—			
	H7	A/Netherlands/219/03	+	0.996	0.016	4.656	—			
IBV	Yamagata	B/Yamagata/16/1988	—				+	0.991	0.141	5.113
		B/Massachusetts/03/2010	—				+	0.993	0.033	4.830

Response: ‘+’ (positive); ‘−’ (negative).

Calibration curves determined by four-parameter linear regression. R^2^ (Coefficient of determination), LOD (Limit of detection), % CV (% Constant of variation).

**Figure 4:**
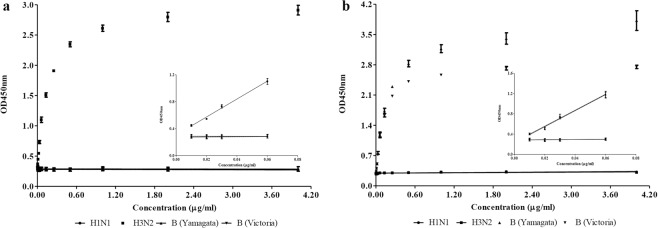
Specificity evaluation of universal antibodies with quadrivalent influenza vaccine components. HAs were the single components of the commercial quadrivalent influenza vaccine supplied by Green Cross pharma (Yongin, Republic of Korea). Error bars indicate standard deviation across 5 replicates. Small graph as an inset represents the linear ranges designated by 4 parameters of linear regression. (**a**) 4F11(group 2 IAV uAb) (**b**) 10F8 (IBV uAb).

Lastly, the uAbs were tested with commercial influenza vaccine lots. Egg-derived influenza quadrivalent vaccine HAs were subjected to the ELISA with 4F11 and 10F8. The ELISA showed that 4F11 bound only to HA from A/Hong Kong/4801/2014 (NYMC X-263B) (H3N2) without cross reactivity to any of the other subtypes: A/Singapore/GP1908/2015 IVR-180 (H1N1), B/Phuket/3073/2013 (Yamagata-like), and B/Brisbane/60/2008 (NYMC BX-35) (Victoria-like) (Fig. 4a). The ELISA respond of 4F11 against H3N2 showed high linearity (R^2^ = 1.000), sensitivity (LOD = 0.003 μg/ml) and reliability (%CV = 3.051). Likewise, 10F8 specifically bound to the HAs from IBV: B/Phuket/3073/2013 (Yamagata-like) and B/Brisbane/60/2008 (NYMC BX-35) (Victoria-like) without any cross reactivity to the HAs from IAV: A/Singapore/GP1908/2015 IVR-180 (H1N1) and A/Hong Kong/4801/2014 (NYMC X-263B) (H3N2) (Fig. 4b). The responds of 10F8 against IBV showed high linearity (R^2^ = 0.999 for B/Phuket/3073/2013 and B/Brisbane/60/2008 (NYMC BX-35)), sensitivity (LOD ≤ 0.006 μg/ml) and reliability (%CV = 3.504 for B/Phuket/3073/2013 and %CV = 3.588 for B/Brisbane/60/2008 (NYMC BX-35)) These results confirmed the group-specific universality of 4F11 and 10F8 to the corresponding influenza HA groups.

In short, we successfully generated group-specific universal antibodies - 4F11 for group 2 IAVs and 10F8 for IBVs - without cross-reactivity to the other HA groups. Both 4F11 and 10F8 effectively bound to the HAs derived from various sources such as embryonated eggs and mammalian/insect cells. In addition, the statistical indicators implied that the response of ELISA using uAb highly correlated with the concentrations of HAs. Furthermore, ELISA can be performed with small amounts of HAs and the results seem to be reliable in repeated tests. However, slight differences were observed in the magnitude of ELISA signals for the HAs, depending on their strain of origin. Whether it is due to structural difference in target epitopes due to sequence variations, or due to an intrinsic difficulty in the measurement of ‘absolute’ amount of HA antigen needs to be addressed for the optimization of the HA quantitative ELISA.

### HA quantification with sandwich ELISA and comparison with the SRID assay

Quantitative sandwich ELISA using uAbs was established to quantify the HAs. The results were compared with those obtained using SRID assay. Reference HA and test HA were pretreated in low pH condition (pH 4.5). Group specific uAbs, 1G5 for group 1 IAVs^15^, 4F11 for group 2 IAVs, and 10F8 for IBVs, respectively, were used as capture antibodies for the ELISA. Strain-specific sheep anti-serum supplied by NIBSC was used as a detection antibody. SRID assay was conducted according to the established standard protocol^8^ and compared with the results obtained using the quantitative ELISA. The antigens representing the individual components for seasonal quadrivalent vaccine produced by Green Cross Pharma (Yongin, Republic of Korea) were diluted to three different concentrations and quantified via ELISA and SRID.

**Figure 5:**
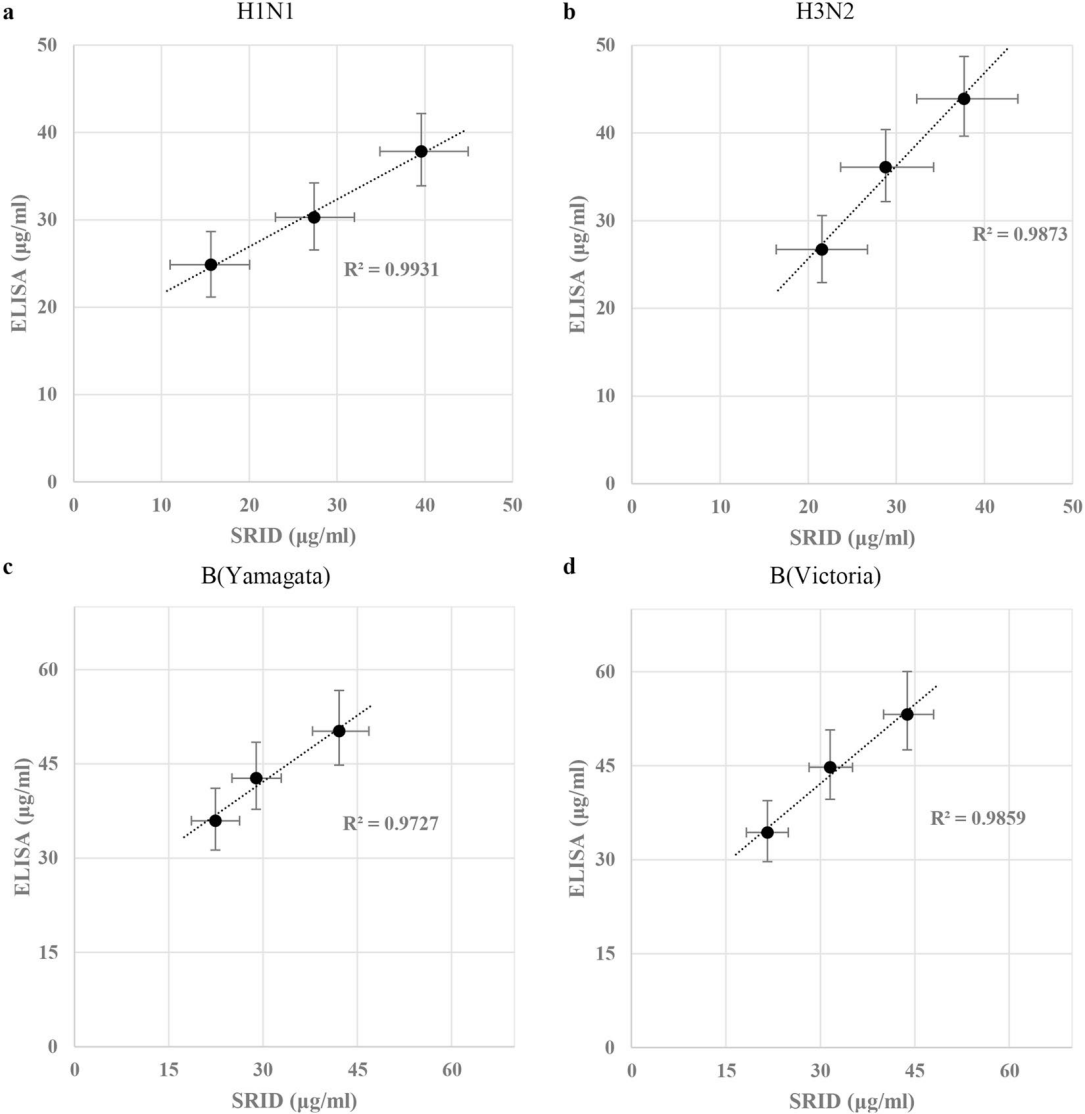
Comparison between ELISA and SRID for the estimation of HA content. The X-axis and Y-axis indicate the estimated concentrations by SRID and by ELISA using uAb, respectively. Error bars indicate 95% confidence intervals across duplicated tests. R^2^ represents coefficient of determination. The HAs subjected to estimations were individual components of quadrivalent influenza vaccine supplied by Green Cross pharma (Yongin, Republic of Korea). (**a**) The quantification of HAs from A/Singapore/GP1908/2015 IBR-180 (H1N1). (**b**) The quantification of HAs from A/Hong Kong/4801/2014X-263B (H3N2). (**c**) The quantification of HAs from B/Phuket/3073/2013 (Yamagata-like). (**d**) The quantification of HAs from B/Brisbane/60/2008 (Victoria-like).

First, the quantification of IAVs (A/Singapore/GP1908/2015 IVR-180 (H1N1) and A/Hong Kong/4801/2014 (NYMC X-263B) (H3N2)) showed that all the estimations using quantitative ELISA could be correlated with those obtained using SRID (R^2^ = 0.9931 for H1N1 and R^2^ = 0.9873 for H3N2 estimation, respectively) (Fig. 5a,b and Supplementary Table 2). SRID results were described in Supplementary Fig. 6. Interestingly, in case of H1N1, the quantification by ELISA tends to over-estimate the potency especially at low concentrations of HA (24.9 μg/ml by ELISA vs 15.6 μg/ml by SRID). This may be caused by higher sensitivity of the ELISA using uAb. In the estimation of H3N2, the ELISA tends to yield a slightly higher value than SRID. Besides, the ELISA results showed smaller error ranges (95% confidence interval) than the SRID results. Therefore, ELISA using uAbs yields results comparable to those obtained by SRID in quantification of HA from IAVs (H1N1 and H3N2).

Furthermore, the quantitation of HA from IBVs (Yamagata-like and Victoria-like lineages) showed that the estimated concentrations of HA by the ELISA correlated well with those obtained by the SRID (R^2^ = 0.9727 for B/Phuket/3073/2013-Yamagata like, and R^2^ = 0.9859 for B/Brisbane/60/2008 (NYMC BX-35)-Victoria like) (Fig. 5c,d and Supplementary Table 2). SRID results were described in Supplementary Fig. 6. Interestingly, all the estimated results by the ELISA were 1.2 to 1.5 times higher than those by the SRID. The sandwich ELISA results revealed that the ELISA responses of the GC flu antigens were higher than those of the NIBSC reference antigens at the same concentrations of the same strains (Supplementary Fig. 7). Similar observations were made in other studies using different set of mAbs^23^. The differences may be caused by the structural difference in quaternary structure of HAs. Alternatively, the degrees of antigen modification introduced during the chemical inactivation procedure (formalin, for instance) might affect the epitopes on the HA and alter the binding characteristics^24–26^. In addition, it may be due to the differences in the measurement of immune complex formation; colorimetric method at a defined wavelength in the case of ELISA, based on the interaction between single molecules of antibody and antigen, *vs* turbidity measurement using the naked eye which becomes distinctive at certain threshold concentration of multiple immune complex in the case of SRID. Certainly, more work is needed for better understanding of the observed discrepancy and further ‘standardization of the standard curve.’

In summary, ELISA using uAb could quantify the HAs in the vaccine preparations and the results were comparable with those obtained with SRID. Further optimization appears necessary to establish more reliable and effective quantitative ELISA protocols.

### HA stability indicating test using universal antibody

The group-specific uAbs were evaluated for their potential for HA stability test. It is generally known that, if a vaccine antigen is exposed to environmental stress such as high temperature or oxidative stress, the structure of immunological relevance could be disrupted, and the potency decreased. To verify if uAbs can bind to structurally disrupted HA or not, HAs (single components of GC flu quadrivalent vaccine), of A/Singapore/GP1908/2015 IVR-180 (H1N1), A/Switzerland/8060/2017 NIB-112 (H3N2), B/Phuket/3073/2013 (Yamagata-like) and B/Maryland/15/2016 NYMC BX-69A (Victoria-like) were exposed to heat stress (60 °C) for various durations (0, 1, 3, 6, 9, 12 hours) and tested by ELISA using uAbs:1G5 for the group 1 IAVs^15^, 4F11 for group 2 IAVs, and 10F8 for IBVs, respectively.

**Figure 6:**
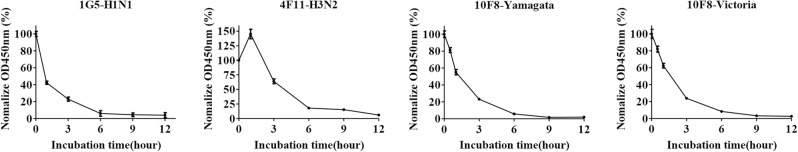
Thermal stability of HA monitored by ELISA using universal antibodies. Each component of quadrivalent influenza vaccine, supplied by Green Cross pharma (Yongin, Republic of Korea), was exposed to 60 °C, and ELISA performed at pre-determined time intervals (0, 1, 3, 6, 9, 12 hours). Error bars indicate standard deviation across 5 replicates. Observed OD_450nm_ were normalized based on the initial OD_450nm_ (0 hour).

Overall, ELISA responses gradually but steadily decreased over prolonged heat exposure (Fig. 6). However, there was an initial, brief increase in the ELISA response, with H3N2 in particular (about 45% in 1 hour). The initial increase in the ELISA response may be related with further exposure of epitopes upon heat stress. Considering the low pH triggered structural changes of HA^15^, which constituted an essential pre-treatment for the present ELISA, the heat stress may further enhance the exposure of the target epitopes in stalk domain for uAb binding, especially for H3N2 HA antigen. Differential sensitivity among independent antigens may be explained by the location of each epitopes recognized by the uAbs. Nevertheless, the decrease in the magnitude of ELISA response over prolonged time of heat exposure observed in all the groups of antigens tested, strongly suggests the utility of the uAbs for testing the stability and the shelf-life of vaccine lots (Fig. 6)

To sum up, ELISA with uAbs may be used for HA stability assay after further optimization of assay conditions. The present work is based on the monovalent vaccine bulk, and further work is needed for trivalent final products for potential interference among the three components and figuring out the best stability-testing module.

## Discussion

The quality assurance of influenza vaccine potency is important for the timely distribution of influenza vaccines. SRID, the only internationally recognized method for checking the potency of vaccines currently, has many limitations. Various alternative experimental platforms for checking the potency of influenza vaccines, including antibody-based assays^27–31^, mass spectrometry^32,33^, HPLC^34–36^, surface plasmon resonance (SPR)^37,38^, and SDS-PAGE^39^, are being developed. Here, we present a new quantitative ELISA for trivalent seasonal influenza vaccine based on the targeting of the conserved stalk domain by HA group-specific uAbs. The consensus HA (cHA) stalk antigens of three components (group 1 and group 2 IAVs and IBVs) were designed *in silico*, expressed as a soluble form in an *E. coli* host, purified^15^ (Supplementary Fig. 3), and used for the generation of mAbs. After immunization, hybridomas were screened for mAbs specific for each group (group 1 *vs* group 2 IAVs) and influenza type (IAV *vs* IBV). The group/type-specific uAbs exhibited specific binding to the HAs – recombinant, egg-derived NIBSC reference materials, and commercial vaccine bulk - in the same group. The sensitivity, linearity, and reproducibility of the ELISA were validated using the NIBSC reference HAs and the recombinant HAs (Tables 1 and 2). This new HA quantitative ELISA method was found to yield results similar to those yielded by SRID (Fig. 5).

Various potency assays using mAbs have been developed as alternatives to the SRID method. Targeting the HA fusion peptide in IAVs^28,30^ may be useful for quantitation of all the HAs of IAV, but cannot differentiate between group 1 and group 2 IAVs, and thus, is unable to quantify individual components of multivalent seasonal vaccine. The strain-specific mAbs^27,29,31^ probably have to be generated against the seasonal drift strains. The mAbs, targeting H1 and H3 components of IAVs, has been recently tailored for the potency assay of multi-component influenza vaccine in microarray format^23^. However, the epitopes of most mAbs are not defined, and therefore, may require repeated tests for their performance for potency assay of seasonal and pandemic vaccines. The present assay based on the stalk domain of HA and the group-specific uAbs is primarily meant to provide an alternative to SRID-based seasonal vaccine potency assay. However, the uAbs targeting the stalk domain could be easily extended to the assays for the pandemic or pre-pandemic mock-up vaccines of H5 viruses of group 1, and H7 viruses of group 2^10,11^. Moreover, the assay could be used for the potency assay of a novel HA stalk-based universal influenza vaccines^12–14^.

For the purpose of wider applications, the referenced subtypes for deducing the cHA stalk of group 2 IAV included both H3 and H7 (Fig. 1); H3 subtypes have been seasonally circulating^40^ since the flu pandemic in 1968^41^, and H7 subtypes are potentially zoonotically transmitted from poultry to humans with a high mortality rate^42^. For IBVs^32^, the stalk sequence homology was extremely high (~98%), and therefore, obviated the need for an *in silico* design of high frequency fragments, as required for IAVs (Supplementary Fig. 1). The specificity of binding among three components (group 1 and group 2 IAVs and IBV) was established for the egg-derived, the mammalian-derived recombinant, and the commercial bulk (Figs. 2 and 3, Supplementary fig. 4 and 5, Fig. 4, respectively). Therefore, the present ELISA format may be used for the potency assay, not only for the traditional egg-derived vaccines, but for the recent cell cultured vaccines^43^ and the novel recombinant HA vaccines^3^. It should be mentioned that the mutation in the stalk region can cause instability of HA trimer and this may be the reason for the ineffectiveness of the H1N1 component of the 2009 pdmH1N1 vaccine^44^. Thus, the HA stalk-based immune response should preferably be assessed to assure the vaccine potency, and the present ELISA using anti-stalk Abs may be suitable for the validation of these factors. A major approach for developing a universal vaccine is to augment the immune responses to the stalk domain, redirecting the response from the variable globular domain^45,46^. A contrasting feature of the present ELISA is that all uAbs are directed to the HA stalk region, and therefore, this assay could serve as a novel potency assay for stalk-based universal influenza vaccines (UIVs)^12^, for establishing correlates of cross-protection^47^, or safety evaluation^48^.

SRID requires strain-specific antisera, and their generation via immunization usually takes 6–8 weeks, sometimes longer. Any delay in this process may lead to hindrance to the timely distribution of influenza vaccines, well exemplified in the 2009 H1N1 pandemic^9^. The newly developed ELISA does not rely on the annual supply of those reagents, and the uAbs can be used independent of HA subtypes of drift strains or pandemic strains. uAbs target the conserved stalk region instead of the polyclonal immune sera directed at the variable globular domain. This would preclude the requirement for generating polyclonal antiserum from sheep against each circulating viruses. Furthermore, it has been acknowledged that the vaccine potency as determined by SRID could not provide an exact correlate between vaccine potency and the clinical outcome^49^. Although the SRID focuses on HA as the major protective antigen, there is growing awareness of the protective role of other influenza antigens including neuraminidase (NA)^50,51^ or HA stalk as additional correlates of protection^7,52^. Thus, the present HA stalk-based assay, in addition to be a powerful alternative to the conventional SRID, can also be extended to establish more clinically relevant assays for testing the vaccine potency.

The guidelines of WHO specify that the vaccine producers must determine the potency at the time of release and throughout the approved shelf life of the product^7^. In this regard, we note that further improvements are needed for the present assay. First, both indirect and sandwich ELISA can be used for the antigen quantification. Sandwich method is generally considered a better choice than indirect ELISA due to higher sensitivity and specificity, but requires additional antibodies either for detection or capture. In this study, we established sandwich ELISA using uAb as capture and strain-specific anti-serum as detection antibodies, respectively. Alternatively, the lineage-specific mAbs directed to the globular domain of IBVs (unpublished results) could be combined with the present uAbs to establish a sandwich-based ELISA for the quantitation of IBVs in the influenza vaccine formulations. Second, the assay tends to overestimate the quantity of HA, especially for IBV components (Fig. 5 and Supplementary Fig. 7). This could be due to the reference antigens developed for SRID, which may not adequately represent the composition of vaccines^53^. Of note, the need for evaluation of critical differences among traditional reference antigens, monovalent bulk materials, final vaccine formulations, and a newer recombinant vaccine, has been raised^23^. Moreover, the performance on the HA stability test needs to be further optimized.

Designed to target the conserved stalk domain, the individual uAbs may differ in their ability to access the target epitopes, which probably are masked by the globular domain in the pre-fusion conformation. In our studies, low pH treatment enhanced the binding of uAbs to HAs by triggering conformational transition that exposed stalk domain^15,54^. Of note, similar acid treatment limited the detection of full potency via SRID based on the strain-specific Abs predominantly targeting the globular domain^55^. Opposing effects of acid exposure may reflect the differences in the assay format and the location of epitopes targeted by antibodies, which merit further investigation. Although various conditions have been tested (such as pH and reducing agents) as influencing factors for the exposure of the stalk domain, more factors need to be evaluated to tailor for stability under stressful conditions.

In summary, we established a prototype ELISA-based quantitative assay system for influenza HA-based group/type-specific universal antibodies for group 1 and group 2 IAVs and IBV. Contingent on further optimization, we offer a potency assay for trivalent seasonal influenza vaccine as an alternative to the SRID. We do note, however, that the present version of ELISA-based method might not be suitable for the testing of quadrivalent vaccines, since the uAb for IBV cannot distinguish the two different lineages of IBVs (Victoria-like and Yamagata-like). Progress is being made to develop lineage-specific uAbs, targeting conserved epitopes in the globular domain that distinguish the HAs from the two lineages of IBVs in quadrivalent vaccine formulation (unpublished results). The repertoire of uAbs against IBVs, along with those of IAVs^15^, could be harnessed for developing and increasing the versatility of the potency assays of seasonal, pandemic, and universal influenza vaccines.

## Materials and Methods

### Generation of consensus HA stalk

HA sequences were obtained from the Influenza Virus Resource in the National Center for Biotechnology Information (NCBI- IVR). The sequence libraries of group 2 IAVs [including H3 (9241 isolates) and H7 (115 isolates)] and IBV (1500 isolates) were established and analyzed using Vector NTI Advanced® version 11.5 (Thermo Fisher, Waltham, MA) and Seq. 2logo 2.0^56^ to deduce consensus HA stalk sequences. Detailed procedures were described in the previous report as applied to group 1 IAVs^15^.

Sequence based secondary structure prediction was carried out by the Network Protein Sequence analysis (NPS@ server SOPMA)^16,17^. Group 2 IAVs and IBVs used for the secondary structure prediction of cHA stalk include A/Texas/50/2012 (GenBank: ALG0759.1), A/Brisbane/10/2007 (GenBank: AIW60702.1), A/Hong Kong/485197/2014 (GenBank: AKS48059.1), A/chicken/Italy/1067/1999 (GenBank: CAE48276.1), A/black duck/Maryland/415/2001 (GenBank: ACD03590.1), B/Yamagata/16/1988 (GenBank: ABL77255.1), B/Malaysia/3120318925/2013 (GenBank: ANK57684.1), B/Massachusetts/02/2012 (GenBank: AGL06036.1), B/Brisbane/60/2008 (GenBank: ANC28539.1), B/Maryland/15/2016 (GenBank: ASW32353.1) and B/Malaysia/2506/2004 (GenBank: ACR15732.1). The secondary structures based on the consensus cHA stalk sequence were compared with those of the viral HA stalks from natural isolates.

### Expression and purification of the cHA stalk

An expression vector with RNA Interaction Domain of lysyl tRNA synthetase from mouse (mRID) was used as the fusion partner^15,18^. The codon-optimized cHA stalk genes for the group 2 IAV and IBV were inserted into the vector.

mRID fused cHA stalk (mRID-cHA stalk) were expressed in *E. coli* BL21 star (DE3) pLysS (Invitrogen, Carlsbad, CA). The *E. coli* was cultured at desired temperature in Luria-Bertani (LB) medium with 1 mM each of ampicillin and chloramphenicol. After cultivation, the cells were sonicated and centrifuged. Total cell lysates (T), soluble (S) and insoluble pellet fraction (P) were analyzed using SDS-PAGE.

Protein purification was carried out using the ÄKTA prime plus chromatography system (GE Healthcare, Chicago, IL) and a HisTrap HP column (GE Healthcare, Chicago, IL). Supernatant that contained the expressed protein was diluted in purification buffer (50 mM Tris-cl (pH 7.5), 300 mM NaCl, 10 mM imidazole, 10% glycerol, 2 mM mercaptoethanol, 0.1% TWEEN^®^-20) and loaded onto a Ni-nitrilotriacetic acid resin column. The proteins were eluted using a linear gradient of imidazole (10–300 mM). Purified mRID-cHA stalks were dialyzed against storage buffer (50 mM Tris-Cl (pH 7.5), 100 mM NaCl, 0.1 mM EDTA, 0.1% TWEEN^®^-20). The final concentrations of the mRID-cHA stalk after concentrating with Centriprep^®^ centrifugal filter (Merck Millipore, Burlington, MA) were determined using densitometric quantitation of Coomassie-stained protein bands obtained by SDS-PAGE and bovine serum albumin (BSA) bands (Amresco, Solon, OH) with known concentrations.

### Generation of monoclonal antibodies

Monoclonal antibodies against the mRID-cHA stalks were generated by murine cell fusion/hybridoma^19^ by ATGen (Seongnam, Republic of Korea). All animal research was performed according to the guidelines of Ministry of Food and Drug Safety of Republic of Korea. All the experiments were approved by ATGen Institutional Animal Care and Use Committee (IACUC; permit number: ATGen2016-0113-06). BALB/c mice were purchased from NARA Biotech (Seoul, Republic of Korea). 8-weak-old female mice were immunized two times at two-weeks interval. Screening for hybridoma clones from the sacrificed mice that were positive to the mRID-cHA stalk protein was done using ELISA. Selected positive clones were purified using Protein G resin (GE Healthcare, Chicago, IL) and dialyzed against PBSA (PBS with 0.05% sodium azide).

### Reference HA antigens

Various HAs were used for the experiments; details of the mammalian-derived recombinant HAs (Sino Biological, Beijing, China), egg-derived reference HAs supplied by the National Institute for Biological Standards and Control (NIBSC, Blanche Lane, UK), and monovalent bulk of quadrivalent seasonal influenza vaccine (GC FLU Quadrivalent) produced by Green Cross Pharma (Yongin, Republic of Korea) are described in Supplementary Table 1.

### Indirect ELISA

The serial two-fold dilutions of HA antigens, which were pretreated with pH 4.5 sodium acetate buffer (NaOAc) containing 200 mM DTT (1,4-dithiothreitol) according to previously established protocol^15^, were coated on immunoassay plates (Nunc-Immuno™ MicroWell™ 96 well solid plates; Thermo Fisher, Waltham, MA) at 4 °C overnight. Then, the plates were blocked with 5% solution of skim milk (BD Diagnostic, Franklin Lakes, NJ) at 37 °C for 1 hour. Next, the uAb was added and incubated at 37 °C for 2 hours. After this, peroxidase-conjugated goat anti-mouse IgG antibody (Sigma-Aldrich, St. Louis, MO) was added and incubated at 37 °C for 1 hour. The plates were washed with PBST buffer (PBS with 0.05% Tween-20) after each step. At last, peroxidase substrate (BD Biosciences, Franklin Lakes, NJ) was added and incubated at 37 °C in the dark for 30 minutes. The substrate development was terminated by adding 0.2 N H_2_SO_4_. A FLUOstar Optima microplate reader (BMG Labtech, Ortenberg, Germany) was used for measuring optical density at 450 nm (OD_450nm_).

### Single radial immunodiffusion (SRID) assay

SRID assay was performed following the established protocol^8^, with minor modifications. The standard HA antigens and the corresponding anti-serum were supplied by NIBSC. The test HA antigens were single components of the quadrivalent influenza vaccine and were supplied by the Green Cross Pharma (Yongin, Republic of Korea). The concentration of test HA antigen was calculated by the slope ratio method^57^ based on the diameters of precipitated circle generated by antigen-antibody reactions.

### Quantification of HA using sandwich ELISA

The reference and test HA antigens were from NIBSC and Green Cross Pharma, respectively. The group specific ‘capture’ antibody against HA stalk was diluted with 0.05 M carbonated-bicarbonate buffer (Sigma-Aldrich, St Louis, MO) and coated on immunoassay plates (Nunc-Immuno™ MicroWell™ 96 well solid plates; Thermo Fisher, Waltham, MA) and kept at 4 °C overnight. The plates were blocked with 5% solution of skim milk (BD Diagnostic, Franklin Lakes, NJ) at 37 °C for 1 hour. Then, the serial two-fold diluted HAs, pretreated with 10% (w/v) Zwittergent 3–14 detergent (Sigma-Aldrich, St. Louis, MO) and pH 4.5 NaOAc buffer^15^, were added and incubated at 37 °C for 1 hour. Anti-HA serum that corresponded with the subjected HA antigen was added and incubated for 1.5 hours. Next, peroxidase-conjugated anti-Sheep IgG H & L (HRP) (ab97130; Abcam, Cambridge, UK) was added and incubated for 1 hour. Subsequent steps were identical with the indirect ELISA. The concentration of test HA was calculated by the slope ratio method^57^ based on the slope of linear regions in the calibration curve of the ELISA response.

### Hemagglutinin stability test

The quadrivalent vaccine HAs were subjected to short-term heat stress (60 °C for 0, 1, 3, 6, and 12 hours). The incubated samples were pretreated with NaOAc buffer (pH 4.5) for 2 hours and assessed via indirect ELISA with corresponding uAbs.

### Statistical analysis

The results of experiments are reported as the mean ± standard deviation with repeated tests. Four-parameter linear regression was implemented using GraphPad Prism version 5.01 (GraphPad Software, La Jolla, CA).

## Supplementary information

Supplementary materials

## Data Availability

All the data from this study are available from the authors after approval of funding bodies.
