# Supplementary material for: Hemagglutinin Quantitative ELISA-based Potency Assay for Trivalent Seasonal Influenza Vaccine Using Group-Specific Universal Monoclonal Antibodies
Source: Sci Rep. 2019 Dec 23;9:19675. doi: 10.1038/s41598-019-56169-5 (PMC6927952; doi:10.1038/s41598-019-56169-5)
Supplement: Supplementary file 1 — Supplementary materials [file 41598_2019_56169_MOESM1_ESM.pdf]

Hemagglutinin Quantitative ELISA-based Potency Assay for Trivalent Seasonal Influenza Vaccine Using Group-Specific Universal Monoclonal Antibodies

Wonil Chae[1,2], Paul Kim[2,3], Hanna Kim[1,2], Yu Cheol Cheong[1,2], Young-Seok Kim[1,2], Sang Moo Kang[4], Baik L. Seong[1,2*]

[1]*Department of Biotechnology, College of Life Science and Biotechnology, Yonsei University, Republic of Korea*

[2]*Vaccine Translational Research Center, Yonsei University, Republic of Korea*

[3]*Department of Integrated OMICS for Biomedical Science, College of World Class University, Yonsei University, Republic of Korea*

[4]*Center for Inflammation, Immunity & Infection, Institute for Biomedical Sciences, Georgia State University, Atlanta, GA, USA*

Supplementary table 1. Reference hemagglutinin antigens.

Supplementary table 2. Comparison of estimated HA concentrations via SRID and ELISA.

Supplementary fig. 1. Sequence of the hemagglutinin stalk.

Supplementary fig. 2. Comparison of secondary structure of HA stalk between the consensus sequence and natural influenza isolates.

Supplementary fig. 3. Expression and purification of mRID-cHA stalk.

Supplementary fig. 4. Evaluation of group 2 IAV universal antibody 4F11.

Supplementary fig. 5. Evaluation of IBV universal antibody 10F8.

Supplementary fig. 6. Quantitation of vaccine HA antigens using SRID.

Supplementary fig. 7. Comparison of the ELISA responses between commercial vaccine and NIBSC reference HAs.

Supplementary table 1

a

Group	Subtype	Strain	Expression host	Cat No.
Group 1 IAV	H1N1	A/California/07/2009	HEK293	11085-V08H
	H2N2	A/Canada/720/2005	HEK293	11688-V08H
	H5N1	A/Indonesia/5/2005	HEK293	11060-V08H1
Group 2 IAV	H3N2	A/Texas/50/2012	Insect cell	40354-V08B
		A/Brisbane/10/2007	HEK293	11056-V08H
	H7N7	A/Netherlands/219/03	Insect cell	11082-V08B
IBV	Yamagata	B/Yamagata/16/1988	Insect cell	40157-V08B
		B/Massachusetts/03/2010	Insect cell	40191-V08B

b

Group	Subtype	Strain	Expression host	Cat No.
Group 1 IAV	H1N1	A/California/7/09 (NYMC-X181)	Egg	16/106
		A/Brisbane/59/2007 (IVR-148)	Egg	08/100
	H2N2	A/Singapore/1/57	Egg	99/714
	H5N1	A/Anhui/1/05 IBCDC-RG-6	Egg	07/290
Group 2 IAV	H3N2	A/Hong Kong/4801/2014 (NYMC X-263B)	Egg	16/286
		A/Switzerland/9715293/2013 (NIB88)	Egg	14/254
		A/Texas/50/2012 (NYMC X-223A)	Egg	13/116
	H7N3	A/mallard/Netherlands/12/2000	Egg	07/336
	H7N9	A/Anhui/1/2013	Egg	16/238
IBV	Yamagata	B/Phuket/3073/2013	Egg	16/158
		B/Massachusetts/02/2012	Egg	13/134
	Victoria	B/Brisbane/60/2008 (NYMC BX-35)	Egg	16/118
		B/Maryland/15/2016	Egg	18/100

(C)

Group	Subtype	Strain	Expression host	Lot No.
Group 1 IAV	H1N1	A/Singapore/GP1908/2015 IVR-180	Egg	V30117122
Group 2 IAV	H3N2	A/Hong Kong/4801/2014 X-263B	Egg	V30117116
IBV	Yamagata	B/Phuket/3073/2013	Egg	V30117069
	Victoira	B/Brisbane/60/2008	Egg	V30117120

(D)

Group	Subtype	Strain	Expression host	Lot No.
Group 1 IAV	H1N1	A/Singapore/GP1908/2015 IVR-180	Egg	V30119008
Group 2 IAV	H3N2	A/Switzerland/8060/2017 NIB-112	Egg	V30118245
IBV	Yamagata	B/Phuket/3073/2013	Egg	V30118126C
	Victoira	B/Maryland/15/2016 NYMC BX-69A	Egg	V30118194B

Supplementary table 1. Reference hemagglutinin antigens. a recombinant HAs (Sino Biological, Beijing, China). **b** egg-derived reference HAs (NIBSC, Blanche Lane, UK). **c** monovalent bulk of quadrivalent seasonal influenza vaccine (GC FLU Quadrivalent) (Green Cross Pharma, Yongin, Republic of Korea)

Supplementary table 2

	A/Singapore/GP1908/2015 IVR-180 (H1N1)					
	SRID			ELISA		
	Lower Limit (µg/ml)	Estimate (µg/ml)	Upper Limit (µg/ml)	Lower Limit (µg/ml)	Estimate (µg/ml)	Upper Limit (µg/ml)
T1	34.9	39.6	44.9	33.9	37.8	42.2
T2	23.0	27.4	32.0	26.6	30.3	34.2
T3	11.0	15.6	20.0	21.2	24.9	28.7

	A/Hong Kong/4801/2014 (NYMC X-263B) (H3N2)					
	SRID			ELISA		
	Lower Limit (µg/ml)	Estimate (µg/ml)	Upper Limit (µg/ml)	Lower Limit (µg/ml)	Estimate (µg/ml)	Upper Limit (µg/ml)
T1	32.3	37.7	43.8	39.7	43.9	48.7
T2	23.7	38.8	34.2	32.2	36.1	40.4
T3	16.4	21.6	26.7	23.0	26.7	30.6

	B/Phuket/3073/2013 (Yamagata-like)					
	SRID			ELISA		
	Lower Limit (µg/ml)	Estimate (µg/ml)	Upper Limit (µg/ml)	Lower Limit (µg/ml)	Estimate (µg/ml)	Upper Limit (µg/ml)
T1	37.9	42.1	46.9	44.8	50.2	56.7
T2	25.1	28.9	28.9	37.8	42.7	48.4
T3	18.6	22.4	26.3	31.3	36.0	41.1

	B/Brisbane/60/2008 (Victoria-like)					
	SRID			ELISA		
	Lower Limit (µg/ml)	Estimate (µg/ml)	Upper Limit (µg/ml)	Lower Limit (µg/ml)	Estimate (µg/ml)	Upper Limit (µg/ml)
T1	40.0	43.8	48.0	47.5	53.2	60.1
T2	28.2	31.6	35.1	39.7	44.8	50.7
T3	18.3	21.6	24.9	29.7	34.3	39.4

Supplementary table 2. Comparison of estimated HA concentrations via SRID and ELISA

Supplementary fig. 1

a

H3 high frequency fragment

321 CPRYVKG--LKLA-GLRNVPEKG-RGIFG-I-GFIENGWE

361 GLVDGWYGFRHGN-EG-GGAADLK--GAAIDGINGKLNRL

401 IGK-NEKFHGIEKEF-EVEGRIGDLEKYVED-KIDLW-YN

441 AELLVALENGH-IDL-D-ELNKLFEK-KKGLRENAEDLGN

481 GCFRIYHKCDNACIG-IRNG-YDHDVYRDEALNNRFGIKG

521 VFLK-GYKDWILWI-FAI-CFLLCVALLGFILWACGKGNI

561 RCNICI

H7 high frequency fragment

314 CPRYVKGR-LLLA-GLKNVPEIPKGRGLFGAIAGFIENGWE

355 GLIDGWYGFRHGNAGGEG-AADYK--G-AIDGI-GKLNRL

395 IEK-NGGFELIDNEFNEVEKGIGNVINW-RD-I-EVW-YN

435 AELLVALENGH-IDLAD-ELDKLYERVKRGLRENAEEDG-

475 GCFEIFHKCDDDCLA-IRNN-YDH-KYREEALGNRIGIDP

515 VKL--GYKDVILWF-FGA-CFILLAIYLGLVFICVKNGNL

555 RC-ICI

Continued at the next page

b

consensus HA stalk (group 1 A virus)

1 GLFGAIAGFI EGGWEGLVDG WYGYHHQNEQ

31 GVGYAADLKS TQNAIDKITN KVNIVIEKLN

61 KQFEAVGKEF NHLEKRIENL NKKVDDGFLD

91 IWAYNAELLV LLENERTLDY HDANVKNLYE

121 KVRLQLKNNA KEIGNGCFEF YHKCDNECLE

151 SYKNGTYDYP KYEEEAKLNR EEIDGVKLEL

181 GVYGIL

Template: H1 high frequency fragment
Red color: score > 0.5 in H1 fragment
Blue: H5 fragment
Green: H9 fragment
Yellow: H2 fragment

Consensus HA stalk (group 2 A virus)

1 GLFGAIAGFIENGWEGLIDGWYGFRHQNAG

31 GEGQAADYKSTQAAIDQINGKLNRLIGKTN

61 EKFHQIEKEFNEVEGRIQDLEKYVEDTKID

91 LWSYNAELLVALENQHTIDLADSELDKLFE

121 KVKKQLRENAEDLGNGCFRIYHKCDNACIG

151 SIRNGTYDHDVYRDEALNNRIGIDPVFLKS

181 GYKDWIL

Template: H3 high frequency fragment
Red color: score > 0.5 in H3 fragment
Blue: H7 fragment

Consensus HA stalk (B virus)

1 GFFGAIAGFLEGGWEGLIAGWHGYTSHGAH

31 GVAVAADLKSTQEAINKITKNLNSLSELEV

61 KNLQRLSGALDELHNEILELDEKVDDLRAD

91 TISSQIELAVLLSNEGIINSEDEHLLALER

121 KLKKLLGPSAVDIGNGCFETKHKCNQTCLD

151RIAAGTFNAGEFSLPTFDSLNITAASLNDD

181 GLDNHTIL

Supplementary fig. 1. Sequence of the hemagglutinin stalk. a High frequency fragment of H3 subtype and H7 subtype. **b** Amino acids sequences of cHA stalks for group 2 IAVs and IBV.

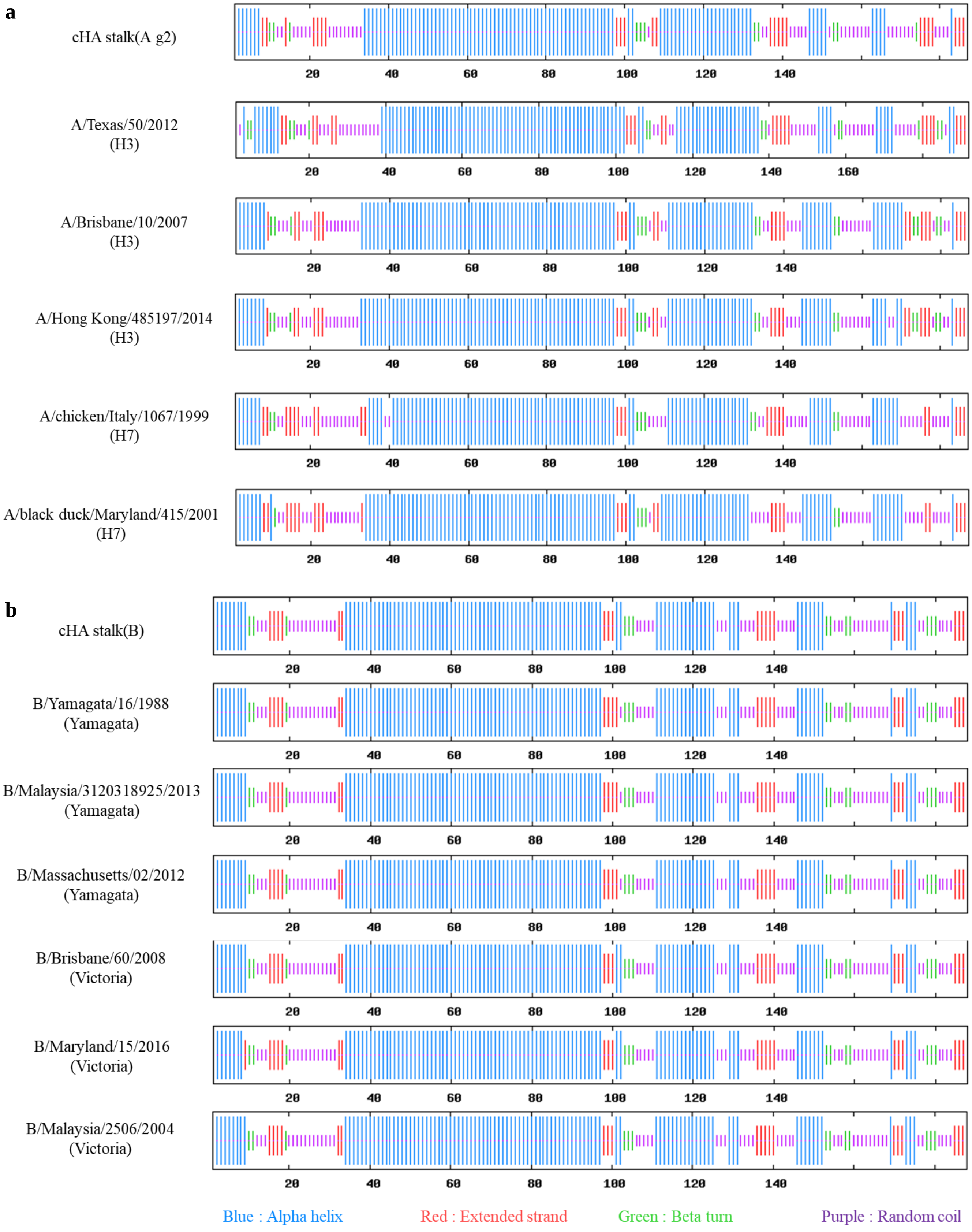

Supplementary fig. 2. Comparison of secondary structure of HA stalk between the consensus sequence and natural influenza isolates. a structures of HA stalks of group 2 IAVs. **b** structure of HA stalks of IBV.

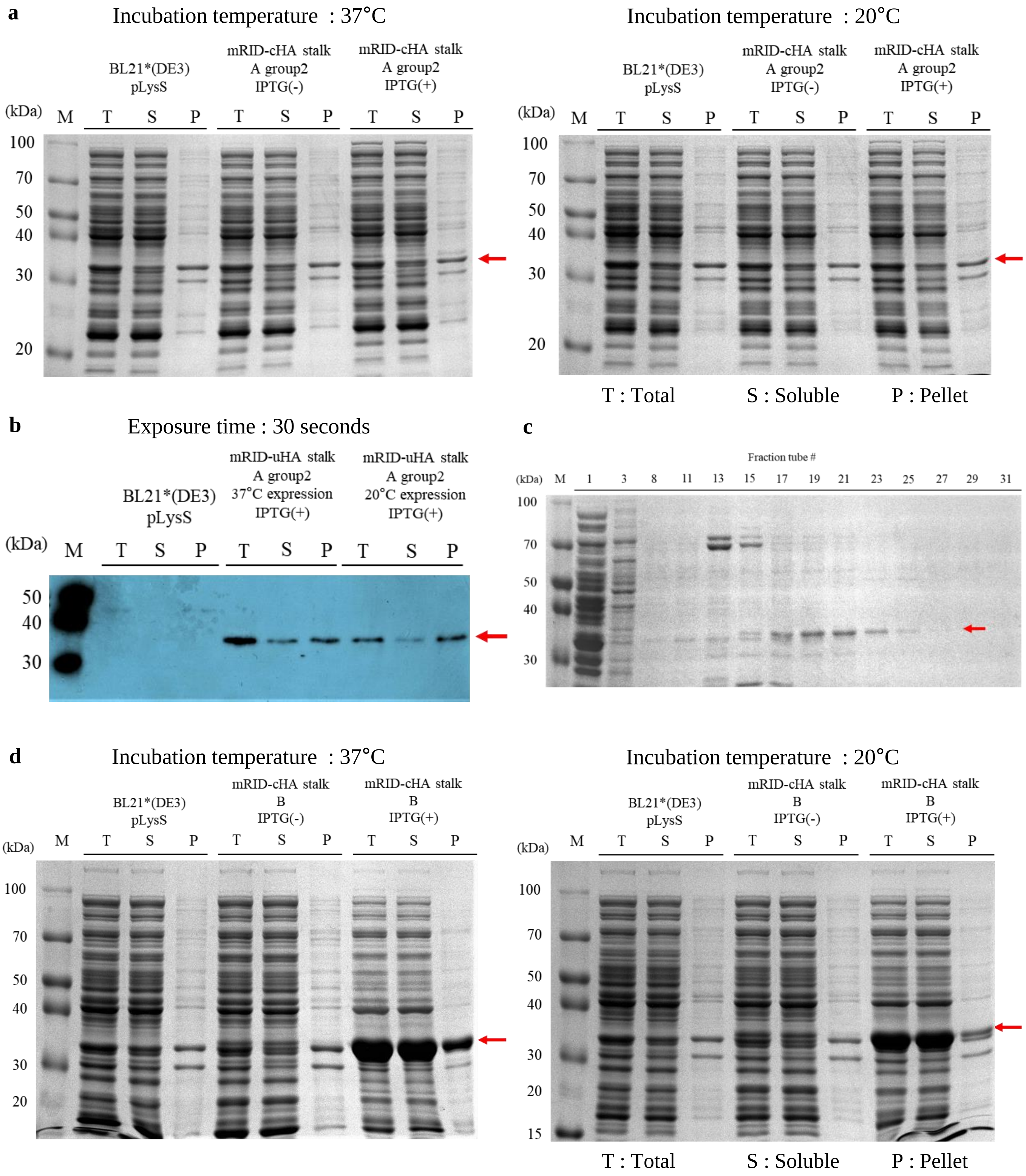

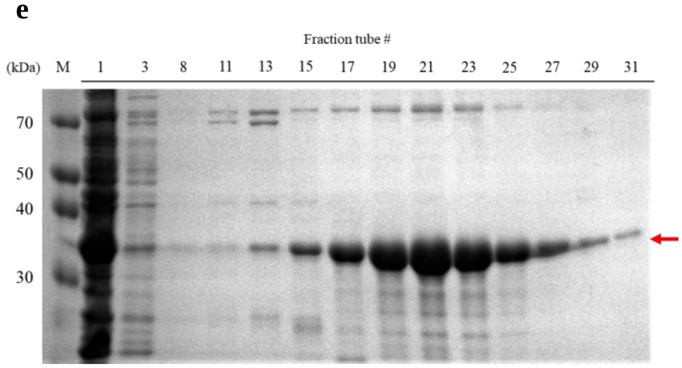

Continued at the next page

Supplementary fig. 3. Expression and purification of mRID-cHA stalk. Red arrows indicate the expressed mRID-cHA stalks protein (~32.2 kDa). ‘BL21*(DE3) pLysS’ section indicates expression profile of *E. coli* cells without transformation. ‘mRID-cHA stalk’ section indicates expression profile of transformed cells. ‘Total (T)’ means total proteins of expressed, ‘Soluble (S)’ means soluble fraction among expressed proteins and ‘Pellet (P)’ means insoluble fraction among expressed proteins. Expression level was examined with inducer (IPTG (+)) and without inducer (IPTG (-)). **a** Expression profile (SDS-PAGE) of mRID-cHA stalk for group 2 IAV. **b** Expression profile (Western blot) of mRID-cHA stalk for group 2 IAV. **c** Purification profile (SDS-PAGE) of mRID-cHA stalk for group 2 IAV. **d** Expression profile (SDS-PAGE) of mRID-cHA stalk for IBV. **e** Purification profile (SDS-PAGE) of mRID-cHA stalk for IBV.

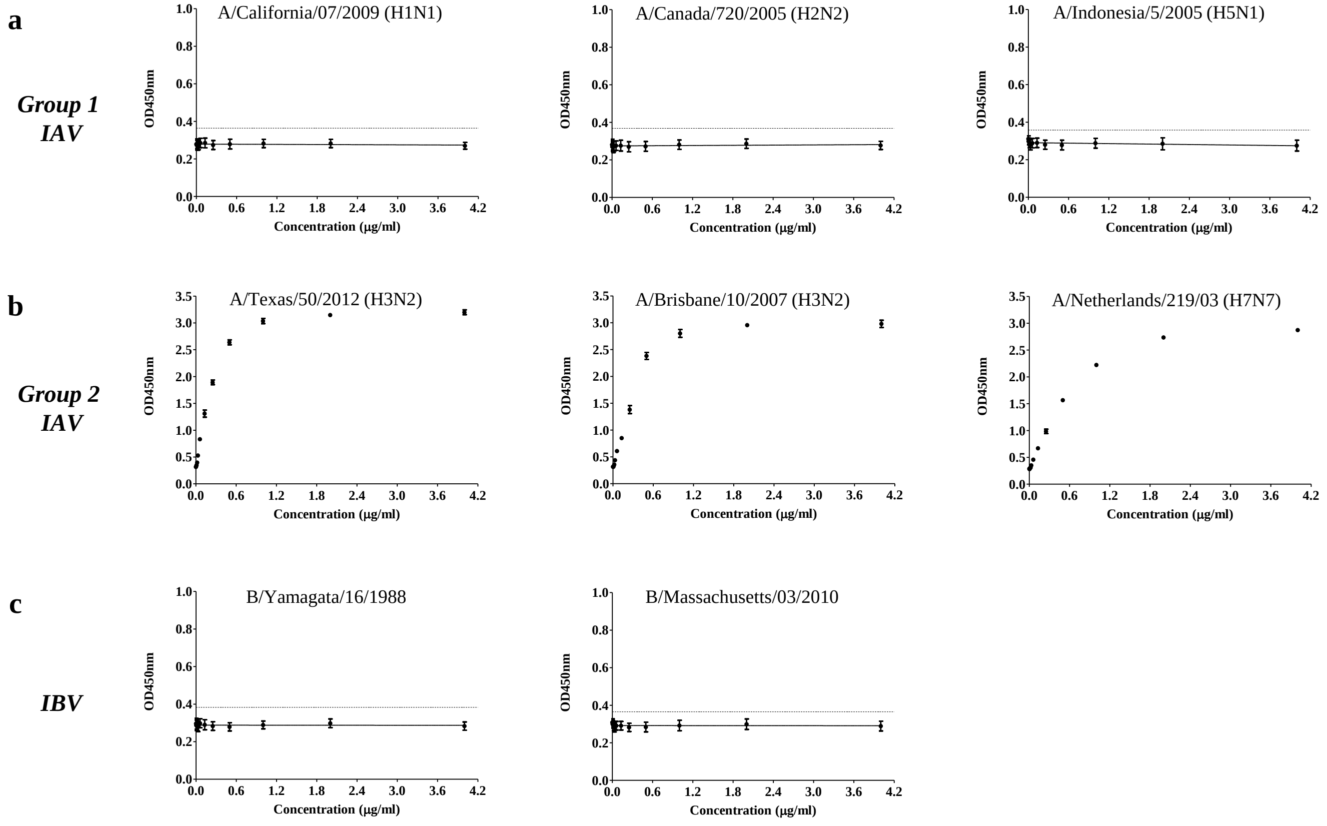

Supplementary fig. 4. Evaluation of group 2 IAV universal antibody 4F11. Group-specific universality of 4F11 was validated by ELISA with mammalian-derived HAs (Sino biological, China). Error bars indicate standard deviation across 5 replicates. Dotted lines indicate limit of detection (LOD = $\text{Mean}_{(\text{PBS})}$ + $3\text{SD}_{(\text{PBS})}$). **a** ELISA with HAs from group 1 IAV. **b** ELISA with HAs from group 2 IAV. **c** ELISA with HAs from IBV.

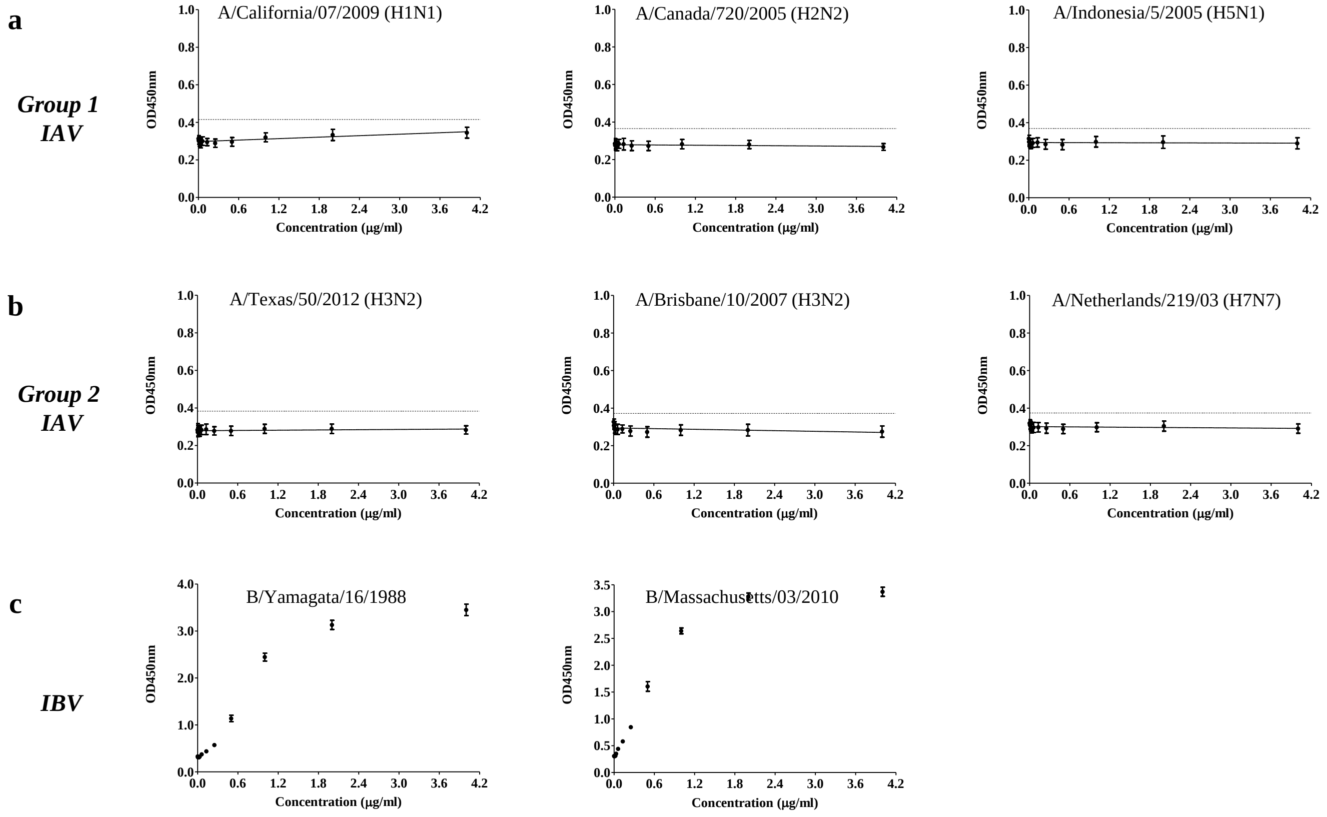

Supplementary fig. 5. Evaluation of IBV universal antibody 10F8. Group-specific universality of 10F8 was validated by ELISA with mammalian-derived HAs (Sino biological, Beijing, China). Error bars indicate standard deviation across 5 replicates. Dotted lines indicate limit of detection (LOD = $\mathrm{Mean}_{(PBS)} + 3\mathrm{SD}_{(PBS)}$). **a** ELISA with HAs from group 1 IAV. **b** ELISA with HAs from group 2 IAV. **c** ELISA with HAs from IBV.

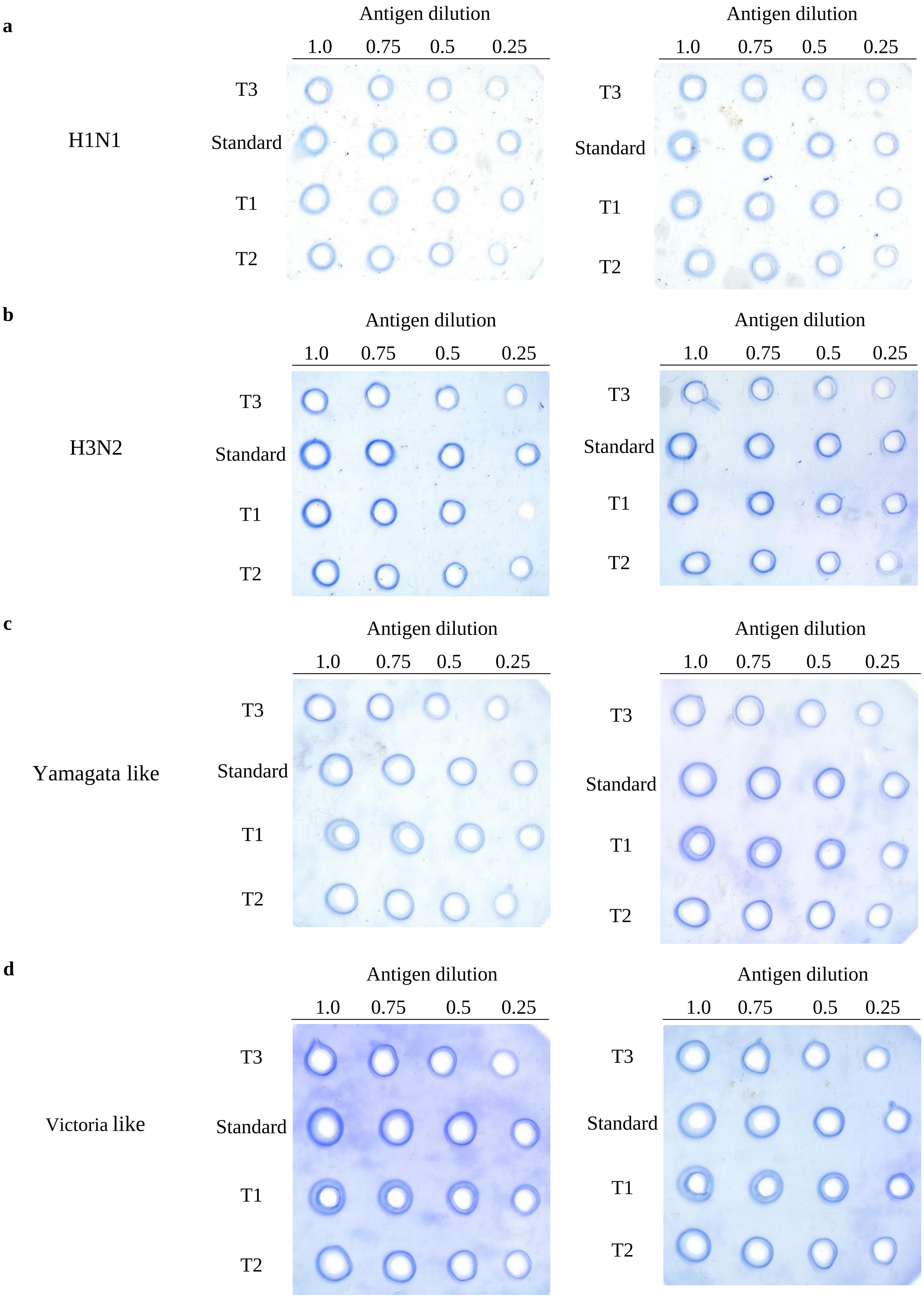

Continued at the next page

Supplementary fig. 6. Quantitation of vaccine HA antigens using SRID. The HAs subjected to SRID were single components of quadrivalent influenza vaccine supplied by Green Cross pharma (Yongin, Republic of Korea) and reference HAs supplied by NIBSC (Blanche Lane, UK). **a** Results of H1N1 HAs **b** Results of H3N2 **c** Results of Yamagata-like HAs **d** Results of Victoria-like HAs.

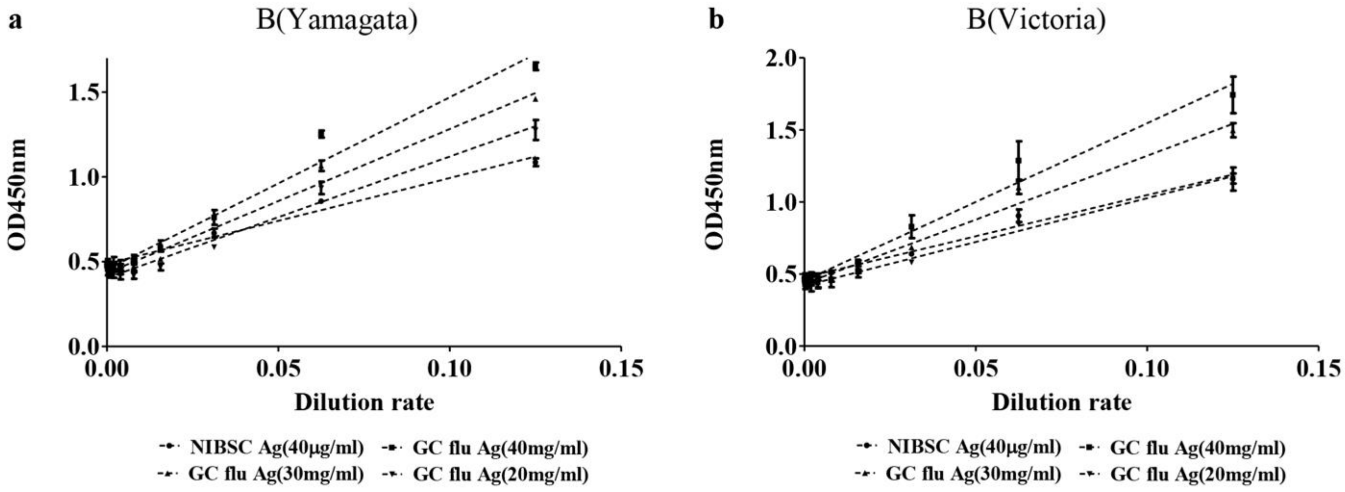

Supplementary fig. 7. Comparison of the ELISA responses between commercial vaccine and NIBSC reference HAs. The HAs subjected to ELISA were single components of quadrivalent influenza vaccine supplied by Green Cross pharma (Yongin, Republic of Korea) and reference HAs supplied by NIBSC (Blanche Lane, UK). Error bars indicate standard deviation across duplicates. **a** ELISA with B/Phuket/3073/2013 (Yamagata-like) **b** ELISA with HAs form B/Brisbane/60/2008 (Victoria-like).
